# The Children’s Respiratory and Environmental Workgroup (CREW) birth cohort consortium: design, methods, and study population

**DOI:** 10.1186/s12931-019-1088-9

**Published:** 2019-06-10

**Authors:** James E. Gern, Daniel J. Jackson, Robert F. Lemanske, Christine M. Seroogy, Umberto Tachinardi, Mark Craven, Stephen Y. Hwang, Carol M. Hamilton, Wayne Huggins, George T. O’Connor, Diane R. Gold, Rachel Miller, Meyer Kattan, Christine C. Johnson, Dennis Ownby, Edward M. Zoratti, Robert A. Wood, Cynthia M. Visness, Fernando Martinez, Anne Wright, Susan Lynch, Carole Ober, Gurjit K. Khurana Hershey, Patrick Ryan, Tina Hartert, Leonard B. Bacharier

**Affiliations:** 1 University of Wisconsin School of Medicine and Public Health, Madison, WI 53706 USA; 2 RTI International, East Cornwallis Road, Post Office Box 12194, Raleigh, Research Triangle Park, NC 27709-2194 USA; 3 Boston University School of Medicine, 72 E Concord St, Boston, MA 02118 USA; 4 Channing Laboratory, Brigham and Women’s Hospital, Boston, MA 02115 USA; 5 Columbia University, Vagelos College of Physicians and Surgeons, New York, NY 10032 USA; 6 Henry Ford Health System, Detroit, MI 48202 USA; 7 Johns Hopkins University School of Medicine, Baltimore, MD 21205 USA; 8 Rho Federal Systems Division, Inc., Chapel Hill, NC 27517 USA; 9 University of Arizona, Tucson, AZ 85721 USA; 10 University of California, San Francisco, CA 94143 USA; 11 University of Chicago, Chicago, IL 60637 USA; 12 University of Cincinnati, Cincinnati, OH 45220 USA; 13 Vanderbilt University School of Medicine, Nashville, TN 37232 USA; 14 Washington University School of Medicine, St. Louis, MO 63110 USA; 15 Department of Pediatrics, School of Medicine and Public Health, University of Wisconsin-Madison, Clinical Science Center-K4/918, 600 Highland Ave, Madison, WI 53792-9988 USA

**Keywords:** Asthma, Birth cohort, Longitudinal study, Development, Allergy, Environment, Children

## Abstract

**Background:**

Single birth cohort studies have been the basis for many discoveries about early life risk factors for childhood asthma but are limited in scope by sample size and characteristics of the local environment and population. The Children’s Respiratory and Environmental Workgroup (CREW) was established to integrate multiple established asthma birth cohorts and to investigate asthma phenotypes and associated causal pathways (endotypes), focusing on how they are influenced by interactions between genetics, lifestyle, and environmental exposures during the prenatal period and early childhood.

**Methods and results:**

CREW is funded by the NIH Environmental influences on Child Health Outcomes (ECHO) program, and consists of 12 individual cohorts and three additional scientific centers. The CREW study population is diverse in terms of race, ethnicity, geographical distribution, and year of recruitment. We hypothesize that there are phenotypes in childhood asthma that differ based on clinical characteristics and underlying molecular mechanisms. Furthermore, we propose that asthma endotypes and their defining biomarkers can be identified based on personal and early life environmental risk factors. CREW has three phases: 1) to pool and harmonize existing data from each cohort, 2) to collect new data using standardized procedures, and 3) to enroll new families during the prenatal period to supplement and enrich extant data and enable unified systems approaches for identifying asthma phenotypes and endotypes.

**Conclusions:**

The overall goal of CREW program is to develop a better understanding of how early life environmental exposures and host factors interact to promote the development of specific asthma endotypes.

## Background

The environment during the prenatal period and in early life is a major contributor to the risk of developing childhood asthma. Birth cohort studies from single research centers have identified individual factors that affect the risk for developing childhood asthma, including exposure to allergens, pollutants, bacteria, psychosocial stress, viral respiratory illnesses and patterns of microbial colonization. Despite such advances, further progress in understanding the root causes of asthma have been hampered by the relatively small size of previous studies that typically lack diverse population and broad assessments of early life exposures. Thus, single cohorts lack the resources to integrate multiple types of complex data to identify the individual and combined effects of environmental factors in asthma development, asthma phenotypes and endotypes. Furthermore, broad and diverse populations and environmental assessments are needed to determine and compare interactions with genetics, effect sizes, mediation and additive effects and dose response relationships, and to identify geographical and temporal differences in exposures and how they impact asthma development across the US. Another notable limitation of individual cohorts with modest sample size is the inability to define prenatal and early life risk factors for infrequent outcomes that are of special clinical significance, such as severe asthma and susceptibility to acute exacerbations. Furthermore, differences in data collection and sample analyses make it challenging to pool, harmonize and integrate findings from different cohorts.

To help overcome these challenges, investigators leading 12 asthma birth cohorts and three additional scientific centers across the U.S. have established the Children’s Respiratory and Environmental Workgroup (CREW) consortium. Collectively, the CREW consortium will identify phenotypes of childhood asthma to develop an understanding of which early life environmental influences are causally associated with these different types of asthma and when their influences are relevant, and to identify targets for future efforts aimed at preventing childhood asthma.

## Methods

### Study overview

CREW is funded as part of the NIH Environmental Influences on Child Health Outcomes (ECHO) program [1]. As such, in addition to pooling data and samples among CREW cohorts, CREW data also will be shared with the larger ECHO program. ECHO consists of 34 initial collaborative awards to birth cohorts or consortia that are investigating effects of early life exposures on five health outcomes in childhood: 1) airway disease (e.g. CREW), 2) obesity, 3) perinatal outcomes, 4) neurocognitive development, and 5) positive health. Enrollment for ECHO is currently underway and the total study population will include about 50,000 families, including the new families recruited during the prenatal period by all ECHO awardees.

CREW consists of 12 individual U.S. birth cohorts and three scientific centers (Table 1). Each of the cohorts was originally established with a major focus on identifying prenatal and/or early life exposures that influence the development of asthma. The initial enrollment for CREW cohorts included nearly 9000 newborns, and approximately 6000 of whom are still being followed. Study designs with enrollment criteria for the individual cohorts have been previously described [2–17]. The investigators of CREW member cohorts have a broad variety of expertise related to early life environmental exposures, allergy and immunology, mechanisms of asthma pathogenesis, geospatial technology, pulmonology, epidemiology, virology, biostatistics, biomedical informatics, genetics, epigenetics and transcriptomics. In addition, there are three scientific centers to provide core services for analyses of the microbiome (University of California, San Francisco), genetics and epigenetics (University of Chicago) and geospatial data analyses (Harvard School of Public Health). The administrative center for CREW is at the University of Wisconsin-Madison, and Rho Inc. (Chapel Hill, NC) is the Coordinating Center for CREW.

**Table 1:** CREW Member Cohorts* and Study Populations

	Initial Enrollment	Location	Population	Recruitment	Cauc/WH	AA	Hispanic	Asian	Other/Missing
CAS	835	Suburban Detroit	General	1987–1989	752 (90%)	22 (3%)	20 (2%)	26 (3%)	15 (2%)
CCAAPS	762	Cincinnati	High risk	2001–2003	579 (76%)	158 (21%)	0 (0%)	2 (0.3%)	23 (3%)
CCCEH	727	Manhattan and Bronx	General	1998–2006	0	291 (40%)	436 (60%)	0	0
COAST	289	Madison	High risk, suburban	1998–2000	243 (87%)	12 (4%)	8 (3%)	0 (0%)	7 (2%)
EHAAS	505	Boston	High risk	1994–1996	370 (74%)	64 (12%)	30 (6%)	28 (6%)	7 (1%)
IIS	482	Tucson	General	1997–2003	280 (58%)	24 (5%)	125 (26%)	0 (0%)	53 (11%)
INSPIRE	1952	Middle Tennessee	General	2012–2014	1269 (65%)	351 (18%)	176 (9%)	0 (0%)	156 (8%)
MAAP	120	Metro Detroit	General	2014–2016	85 (71%)	22 (18%)	0 (0%)	4 (3%)	9 (8%)
TCRS	1246	Tucson	General	1980–1984	822 (66%)	50 (4%)	312 (25%)	0 (0%)	62 (5%)
URECA	609	NYC, St Louis, Baltimore, Boston	High risk, urban	2004–2006	22 (4%)	429 (70%)	116 (19%)	0 (0%)	42 (7%)
WHEALS	1258	Metro Detroit	General	2003–2007	325 (26%)	787 (63%)	81 (6%)	30 (2%)	35 (3%)
WISC	212	Rural WI	Rural: farm and non-farm	2013–2019	204 (96%)	4 (2%)	2 (1%)	2 (1%)	0 (0%)
Total	8997				4951 (55%)	2214 (25%)	1306 (15%)	92 (1%)	409 (4%)

*Abbreviations: CAS, Children’s Asthma Study; CCAAPS, Cincinnati Childhood Allergy and Air Pollution Study; CCCEH, Columbia Center for Children’s Environmental Health Cohort; COAST, Childhood Origins of Asthma study; EHAAS,; IIS, Infant Immune Study; INSPIRE, Infant Susceptibility to Pulmonary Infections and Asthma Following RSV Exposure study; MAAP, Microbes, Allergy, Asthma and Pets study; TCRS, Tucson Children’s Respiratory Study; URECA, Urban Environment and Childhood Asthma study; WHEALS, Wayne County Health Environment Allergy and Asthma Longitudinal Study; WISC, Wisconsin Infant Study Cohort

**Fig. 1:**
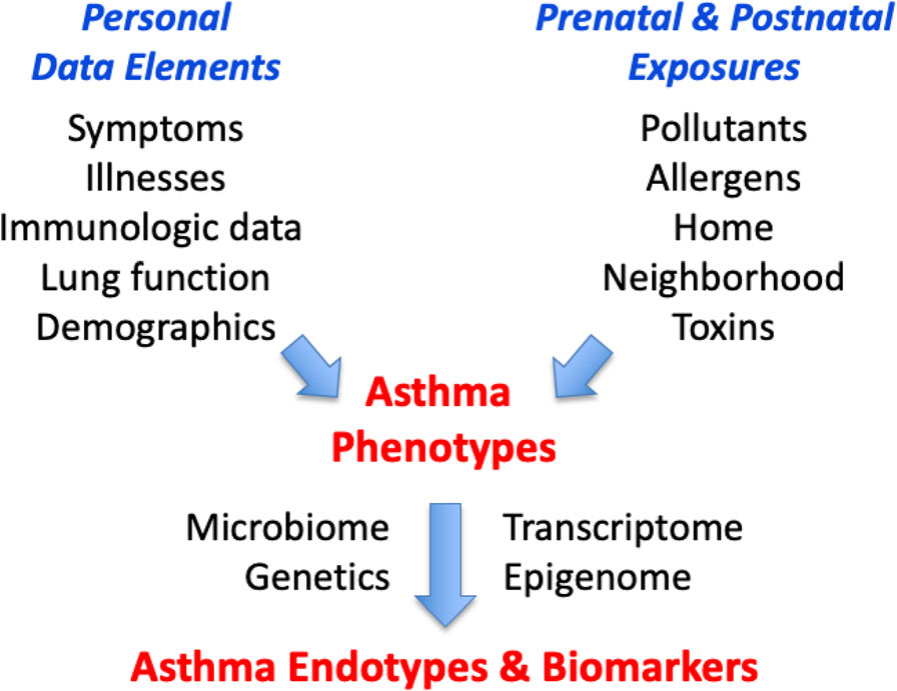
CREW conceptual diagram

CREW has three main hypotheses (Fig. 1). First, longitudinal analyses of individual characteristics, including patterns of respiratory symptoms, aeroallergen-specific IgE, lung function, sex and ethnicity can be used to identify respiratory phenotypes, including those that are highly enriched for asthma. We further propose that these phenotypes will have closer associations with etiologic factors (e.g. environmental exposures, genetics), compared to a general yes/no definition of childhood asthma due to reduction in misclassification. Second, host factors (e.g. genetics) interact with environmental exposures during the prenatal period and early childhood, leading in some cases to epigenetic alterations, and ultimately to specific changes in immune and lung development that increase the risk for specific asthma endotypes. Finally, molecular biomarkers can be identified to provide a precise and comprehensive identification of childhood asthma endotypes.

To address these hypotheses and goals, the CREW consortium has designed three protocols. The first protocol (Phase I) pools extant data from the 12 cohorts to address specific hypotheses. Because the studies were designed by different groups with their own unique objectives, extensive data harmonization will be required. The second (Phase II) protocol will collect new data on current participants from 10 of the cohorts using standardized procedures and questionnaires and will develop standardized outcomes within CREW (and ECHO). Because most of the early life specimens for current CREW participants already have been collected, a third protocol (Phase III, or “NewCREW”) initiating a new multisite cohort will allow standardized collection of prenatal and early life environmental and immunologic samples to measure factors that could influence or be related to the development of allergic diseases and asthma.

### Phase I: leveraging extant data

#### Data sharing agreements

For Phase I, each participating institution reviewed and approved a consortium and data sharing agreement that enables within-group (including the ECHO data analysis center) sharing of previously collected data under a waiver of consent. This agreement was approved by Human Subjects Committees at each of the participating institutions.

Each of the CREW member cohorts has collected data and samples related to personal and environmental characteristics that could affect allergic diseases and asthma, which varied to some degree among cohorts. This information includes clinical questionnaires and biological measures that characterized environmental exposures (e.g. allergens, pollutants and microbes), family and personal history of allergic diseases, respiratory infections and illnesses, immune development, genomic data, and microbial colonization.

The individual datasets have been stripped of protected health information (PHI) as defined by HIPAA, except for those indirect identifiers permitted in a limited data set, such as dates of birth, dates of data and sample acquisition, and town/city, state and zip code. The current Data Sharing Protocol also allows for sharing of this pooled limited dataset with the ECHO Data Analysis Center to enable collaborative research studies with any of the 72 other ECHO cohorts.

#### Data harmonization and analysis projects

Demographic and other exposure and outcome data have been and will continue to be compiled by the individual cohorts and sent to the University of Wisconsin (UW). A Publications Committee has been established to review and approve analysis concepts and resulting publications. Data harmonization is a critical function that allows merging data from the 12 cohorts. The harmonization process requires understanding the original data definitions provided by cohort investigators and data managers, and mapping them into a common CREW definition developed by informaticians. This procedure is performed by data scientists at UW working closely with cohort investigators and data managers.

#### Geospatial approach

Exposures to environmental toxicants and community characteristics during the prenatal period and early childhood have a significant impact on allergic disease and asthma. CREW investigators have developed distributed methods to derive environmental exposures and community characteristics, including measures of disparities, for all CREW participants based on residential locations. A containerized software platform was developed to enable individual CREW sites to geocode their participants’ birth record addresses and link these to community characteristics [18]. Census tract-level information regarding race, ethnicity, education, housing, and other indicators of disadvantage/advantage was obtained by collaborators at Harvard Center for Geographic Analysis (Cambridge, MA) and the University of Arizona using the decennial U.S. census in 1980, 1990, 2000, and 2010. These data, combined with a TIGER/Line address range geocoder, census tract boundaries, and analysis code were containerized and distributed to individual CREW sites. Using this software, each study site can geocode their participants’ birth record address and link these to census-tract level information for further analysis.

### Phase II: standardized data collection

The CREW Phase II protocol includes standardized measures of exposure assessments, immunologic studies, lung function testing and outcome measures. The measurements will be obtained at least once per life stage interval (1–4 years, 5–7 years, 8–10 years, 11–14 years, 15–17 years and 18 years and over) to standardize measurements while accommodating different schedules of the cohorts.

#### Enrollment criteria and recruitment procedures

Participating families from 10 of the CREW member cohorts are eligible for Phase II study enrollment. Exclusion criteria include past or current medical problems that could pose additional risks from participation in the study, interfere with the participant’s ability to comply with study requirements or otherwise adversely impact the quality or interpretation of the data. Recruitment techniques varied by study site, but study coordinators generally provided information about CREW and an invitation to participate during usual cohort activities by phone, email, mail, or at in-person study visits. For cohorts lacking upcoming activities, sites used mailings, secure web-based communication or phone calls to provide information and an invitation to join CREW. For recruitment of adult participants, sites had the flexibility to contact them first via mail or email to gauge interest in participating in CREW. This included a short questionnaire to assess current allergies and asthma, and an invitation to join the new study.

Individual site Human Subjects Committees approved the CREW Phase II protocol. Families or adult participants provide informed consent and children between the ages of 7 and 18 years provide assent before being enrolled in CREW and ECHO.

#### Questionnaires

Questionnaires will be administered to assess the clinical, environmental and medication history of the participants (Table 2). Questionnaire administration will include paper questionnaires, online questionnaires, electronic case report forms (eCRF), and interviewer-administered questionnaires. The study’s primary source of information on the occurrence and frequency of wheezing is the Airways Outcomes questionnaire. This questionnaire includes questions from the ISAAC study [19], supplemented by additional questions related to food allergy, asthma medication use, anaphylaxis and allergic rhinitis. The Airways Outcomes questionnaire (Online data supplement) will also be administered by other ECHO cohorts, to enable standardized outcome assessments for allergic diseases and asthma across all ECHO cohorts. Additional questionnaires will include Tanner staging, parental or self-assessment of atopic dermatitis (POEM [20, 21]), and demographic information, among others (Table 3).

**Table 2:** Study Activities in the CREW Standardized Data Collection Protocol*

	Age (in years)					
	1–4	5–7	8–10	11–13	14–17	> 18
Visit window	+ 12 mon	+ 12 mon	+ 12 mon	+ 12 mon	+ 12 mon	+ 12 mon
Time	2.0 h	3.0 h	3.0 h	3.0 h	3.0 h	3.0 h
Essential procedures						
Consent/Assent	x	x	x	x	x	x
Eligibility	x	x	x	x	x	x
Distribute home collection kits	x	x	x	x	x	x
PE and anthropomorphics	x	x	x	x	x	x
Questionnaires	x	x	x	x	x	x
Blood	x	x	x	x	x	x
Urine	x	x	x	x	x	x
Stool	x	x	x	o	x	o
Nasal wash/blow/lavage	w	b	l	l	l	l
Home dust collection	x					
Spirometry			x	x	x	
Spirometry w/ reversibility		x	o	o	o	x
eNO		x	x	x	x	x
AE collection	x	x	x	x	x	x
Optional procedures						
Nasal brushing	o			o		o
Prick skin tests	o	o	o	o	o	o
Phone call (Follow up)	x	x	x	x	x	x

Abbreviations: w, nasal wash; b, nasal blow; l, nasal lavage; o, optional

**Table 3:** Standardized Questionnaires and Timing of Administration

	Age in years					
Questionnaire	1–4	5–7	8–10	11–13	14–17	> 18
Demographics						
- Addresses	x	x	x	x	x	x
- Household census						
- SES						
Asthma control						
- ACT or Pediatric ACT		x	x	x	x	x
Self-Tanner Stage			x	x	x	
Health history						
- Allergic disease and asthma	x	x	x	x	x	x
- Allergic rhinitis						
- Atopic dermatitis						
- Food allergies						
- Perinatal (if not prospectively recorded)						
POEM (for atopic dermatitis)	x	x	x	x	x	x

#### Physical examination

Physical examination will include anthropomorphics and measurement of heart rate and blood pressure.

#### Blood samples

Peripheral blood will be being collected as fasting morning samples when possible, especially between ages 8–17 years to enable measurement of sex hormones, insulin and glucose. Approximately 15 mL will be obtained at each annual clinic visit. These samples will be transported to the center’s laboratory on the day of collection and at room temperature. At the center-specific laboratories, blood will be processed and aliquots of serum and plasma will be cryopreserved at − 80 °C. In addition, peripheral blood mononuclear cells will be separated from heparinized blood and cryopreserved in aliquots using pre-chilled alcohol reservoirs. A complete blood count and differential will be performed, and plasma will be analyzed for allergen-specific IgE (Uni-CAP, ThermoFisher). DNA from peripheral blood samples will be stored for studies of epigenetic regulation.

#### Airway samples

Nasal wash, blow or lavage specimens will be collected using techniques that are tailored to the age of the child. For young children (birth to age 3–4 years), we will collect a nasal wash specimen by instilling 2 ml of buffered saline into the nose using a modified bulb syringe, followed by gentle suctioning is applied to recover the sample [22]. For capable preschool children, we will use a nasal blow technique [23]. The procedure involves wetting the nose with normal saline spray, followed by blowing the nose into a collection tube.

For adults and children over the age of 8 years, a nasal lavage will be performed. Following a nasal blow while in a sitting position, saline is introduced into the one nostril, and nasal lavage fluid is passively recovered from the other nostril. This is repeated on the other side.

For each of these nasal samples, separate aliquots will be prepared for analyses of virology, microbiome, host cell gene expression, proteins and other analytes.

Nasal epithelial cells will be obtained brushing the inferior turbinate of one nasal passage using a nylon flocked swab. The epithelial cells will be a source for mRNA and DNA (epigenetics) isolation.

#### Other samples (stool, urine, saliva)

The participant will be asked to provide stool samples to examine fecal bacterial and fungal content. The sample can be collected during the clinic visit or at home using the collection kit and directions. If the specimen is collected at home, it can be mailed to the laboratory or brought to the clinic on the day of the study visit. Clean catch urine will be collected for measurement of chemical exposures, inflammation or biomarkers that might be related to asthma. Home collection kits will be distributed during the consenting visit. Pre-packaged collection kits and directions will be given to the participant and/or parent/guardian for collection of stool and urine. The study staff will review the directions with the participant and/or parent guardian.

#### Allergy skin testing

Prick skin testing to identify aeroallergen sensitization using a Multi-Test PC device (Lincoln Diagnostics, Decatur, IL) is an optional procedure at each of the life stages. Indoor and outdoor allergens that were selected for local prevalence (Table 4, Greer Laboratories). Wheal sizes are measured after 15 min. The wheal’s longest length and width (measured perpendicular to the length at its midpoint) are measured to the nearest millimeter and averaged to give a mean wheal size. A positive reaction is defined as a mean wheal size at least 3 mm larger than the saline control.

**Table 4:** CREW skin test antigens

Test #	Extract
1	Control, 50% (V/V) glycerin
2	Histamine 10 mg/ml
3	Mouse epithelia
4	Dog epithelia
5	Cat hair (standardized)
6	*D. farinae* + *D. pteronyssinus* mix
7	American/German cockroach mix
8	*Penicillium notatum*/*Penicillium chrysogenum*
9	*Alternaria tenuis*
10	*Cladosporium herbarum*
11	*Aspergillus* mix
12	Ragweed mix (giant and short)
13	Eastern 6 tree mix (American beech, eastern cottonwood, red oak, red/river birch, shagbark hickory, white ash)
14	K-O-T grass mix (Kentucky blue/june, orchard, timothy)
15	Maple/box elder mix
16	3 weed mix (cockleburr, lamb’s quarter, pigweed)

#### Total and allergen-specific IgE

Total and allergen-specific IgE antibodies are measured by fluoroenzyme immunoassay (ImmunoCAP, ThermoFisher, Uppsala, Sweden). The panel of specific IgE antibodies vary by age and in some cases region of the country. For detection of sensitization in early childhood, the specific IgE panel includes egg white, milk, peanut and a multiple aeroallergen screen (Phadiotop, ThermoFisher). If the Phadiotop is positive, a panel of aeroallergen-specific IgE (cat, dog, mold mix, and house dust mite mix) is measured. For adults and children over the age of 5 years, aeroallergen testing includes birch or oak, ragweed, and Timothy grass; and *Dermatophagoides farinae*, *Dermatophagoides pteronyssinus,* dog epithelium, cat dander/epithelium, German cockroach, mouse urine protein, and *Alternaria alternata*. Measurement of IgE antibodies to selected allergens will be performed at each life stage.

#### Lung function and exhaled nitric oxide (eNO)

To provide standardized protocols for lung function measurement, equipment, and overreading of studies, CREW has established a Pulmonary Function Core. Equipment was selected for spirometry (Jaeger Sentry Suite, Mettawa IL) and measurement of eNO (NIOX VERO®, Circassia Pharmaceuticals Inc., Morrisville, NC). Computer software is loaded on spirometers at each site with onsite training available to facilitate secure transmission of studies to the central server. CREW coordinators attended a 2-stage training session for introductory and advanced spirometry. All lung function studies will be overread by an experienced respiratory technician in Madison.

Beginning at age 5–7 years, children will perform a test for measurement of eNO. Prior to spirometry, exhaled NO is measured employing a technique modified after Silkoff et al. [24] and following American Thoracic Society guidelines for eNO assessment [25]. In brief, this technique utilizes a resistive device that provides a constant low expiratory flow rate and ensures velum closure.

Spirometry will be conducted during clinic visits at each life stage beginning at 5–7 years according to ATS guidelines [26]. Participants will be asked to hold albuterol for 4 h prior to this procedure, and long-acting beta agonists for 12 h before the visit. If the participant feels they need to take their medication because their symptoms get worse, they will be instructed to do so and call the study staff.

Reversibility following administration of albuterol (4 puffs) will be performed at ages 5–7 years of age and in adults (≥ age 18 years) and is optional for intermediate age groups. After completion of spirometry as described above, albuterol via MDI with spacer will be administered. Fifteen minutes later the spirometry will be repeated. In females ≥ age 12 years, urine pregnancy tests will be conducted to ensure that and this reversibility test will not be performed in pregnant woman.

#### Investigator training

Face-to-face and web-based training sessions were held for sample collection and processing and to review questionnaires and recruitment procedures. The initial face-to-face training occurred in December 2017 at the University of Wisconsin-Madison, and included study coordinator and lab representatives from each of the clinical centers. Sessions were designed to cover clinical procedures relating to collection and processing of biological samples, as well as pulmonary function testing, exhaled nitric oxide testing, and consenting. After completion of the in-person training, we conducted additional web-based training sessions during bi-weekly coordinator calls. These sessions are recorded for new staff to review in the future for certification. Web-based training is ongoing and occurs regularly as issues and concerns arise.

CREW employs a comprehensive manual of procedures that cover all aspects of study management and protocol implementation. The training program includes a mixed methodology approach, an accountability component, and pre-recorded training modules to ensure standardization of the training process. To assess training effectiveness, skills are evaluated with written exams, by observation, or certification with a qualified trainer, and by on-going quality control evaluations. Training completion is tracked using a web application (Moodle, https://moodle.org), which is monitored regularly by the Coordinating Center.

### Phase III: recruitment of additional families

The CREW phase III protocol (“NewCREW”) will enroll approximately 400 additional pregnant women and their infants during the prenatal period at four CREW centers. The purpose of this protocol is to collect early life exposure, biospecimens, and outcomes data for the CREW and ECHO protocols. There will be an emphasis on collection of maternal specimens during the prenatal period and extensive pre-, peri- and postnatal environmental sampling. The sampling strategy was designed to supplement and enrich existing data in CREW and ECHO and will provide specimens that are optimized for analyses of early life environmental exposures (e.g. microbial exposures and colonization), immune development (gene transcription, metabolites and proteins), epigenetics (nasal cells) and outcomes (e.g. wheeze, growth, allergic sensitization). Outcomes data will be collected for allergic diseases and asthma, and other ECHO outcomes including perinatal outcomes, neurocognitive development, obesity and positive health.

### Collaboration with the ECHO data collection protocol

The primary objective of the ECHO-wide Cohort Data Collection Protocol is to facilitate the creation of a data platform consisting of essential data elements from all participating ECHO awardees, plus recommended data elements from subsets of cohorts. This protocol standardizes new data collection across the ECHO Program and specifies what data elements cohorts should collect (new data) and share (both existing data and new data) across the life course, from the prenatal period through adolescence, to create the ECHO-wide Cohort. CREW is participating in the ECHO-wide Cohort Data Collection Protocol in both Phases II and III by collecting essential and many recommended data elements. This includes collection of additional biological specimens, such as hair and toenails, for evaluation of chemical exposures.

### Data management

#### Central database

All 3 phases of CREW will receive support from the CREW informatics team in data collection, data management, and the creation of a central database (CREW Asthma eLab) for the study. Asthma eLab was initially developed by investigators at the University of Manchester for use in the STELAR birth cohort consortium [27]. It serves as a secure database, a forum for investigator interaction, and a platform for analyses and manuscript development. With assistance from the NIH, the STELAR group transferred an updated version of Asthma eLab to the UW-Madison, and are helping with software customization, installation and training. STELAR and UW investigators collaborated to create the state-of-the-art Fast Health Interoperability Resources (FHIR) profiles needed for the Asthma eLab platform. These profiles will enable CREW data, research collaborations, data harmonization and data analyses to occur within eLab.

#### Data handling and security

We have developed workflows and infrastructure to support secure data collection for different parts of the CREW project. Electronic clinical research forms (CRFs) were designed and implemented in REDCap to document consent for the Phase II CREW protocol, and a dashboard was implemented to track recruitment. Using a similar approach, about 30 CRFs were implemented to support data collection for the Phase II protocol. An Information Governance Policy was written and approved by the CREW Steering Committee to provide detailed rules for data access, handling and security.

#### Quality control

Quality monitoring procedures for new data collection activities have been established for CREW and start with comprehensive training and certification of all staff before performing any study procedures. All CREW study data are subject to comprehensive built-in edit checks and manual queries as part of the REDCap data entry system. The CREW Coordinating Center employs a suite of statistical data checks to examine data for outliers and anomalies. Standard and custom reports and dashboard visualization displays (e.g. recruitment status, sample explorer tool) are used to monitor data quality and study performance and will be available to all CREW research staff via the CREW password-protected website (RhoPORTAL). These procedures will be implemented with each of the CREW protocols. In addition, we will implement and adhere to all quality monitoring procedures that will be established for the ECHO Data Collection Protocol.

### Computation and statistical analyses

#### The CREW Bioinformatics and Biostatistics Core (BBC)

The BBC was formed to conduct and oversee key computational and statistical analysis tasks. These include establishing and operating the CREW e-Lab system, as described in the previous section. Analytic tasks include identification of the predominant asthma phenotypes and endotypes in the subject population using the high-dimensional, heterogeneous data (symptoms, lung function measures, immunologic variables, etc.). In addition, the BBC will develop models and statistical approaches to identify relationships among key variables in the complex data sets, including measures and load of exposure, epigenetic modifications, transcriptional profiling, metabolomics, protein expression and asthma phenotypes. These models will include longitudinal measurements, assessment of age dependency and consideration of covariates, effect modifiers and mediators.

Because there is a wide range of methodologies that can be applied to these tasks, and the CREW team encompasses a broad range of data-science expertise, we anticipate that we will investigate, develop, and evaluate a variety of approaches for each task. In broad terms, some of the methodologies we will investigate include unsupervised learning, supervised learning, causal modeling, spatial modeling, and hypothesis testing. The BBC is comprised of biomedical bioinformaticists and statisticians from each site of our consortium. This team approach has the advantages of including investigators who are intimately familiar with the data sets while bringing a broad range of analytic expertise to the group.

## Results

### Phase I: leveraging extant data

#### Study population

The study participants of the CREW consortium represent a diverse national sample of children and their families recruited over the past 30 years into the 12 member cohorts. The total combined study population is 8997 at the time of birth (Table 1). The study population is quite diverse in terms of current age (ages < 1 through 36 years), date of recruitment (1980–2018), race and ethnicity. There is also considerable variation in the geographic locations of study participants, with representation from the East Coast (Baltimore, Boston, New York City), Midwest (Cincinnati, Detroit, Madison, Marshfield), South (Nashville, St. Louis) and West (Tucson). In addition to including large numbers of urban participants living close to academic medical centers, the CREW study population also includes suburban (CAS, COAST, EHAAS, WHEALS, INSPIRE) and rural (INSPIRE, WISC) children. The study designs include those selected to represent a general population, high risk cohorts on the basis of parental history of allergies or asthma, and stratified populations based on pet ownership (MAAP) or residence on a dairy farm (WISC). Collectively, the CREW consortium represents the largest and most diverse U.S. longitudinal cohort of children evaluated for asthma (Table 1).

#### Available data elements

**Fig. 2:**
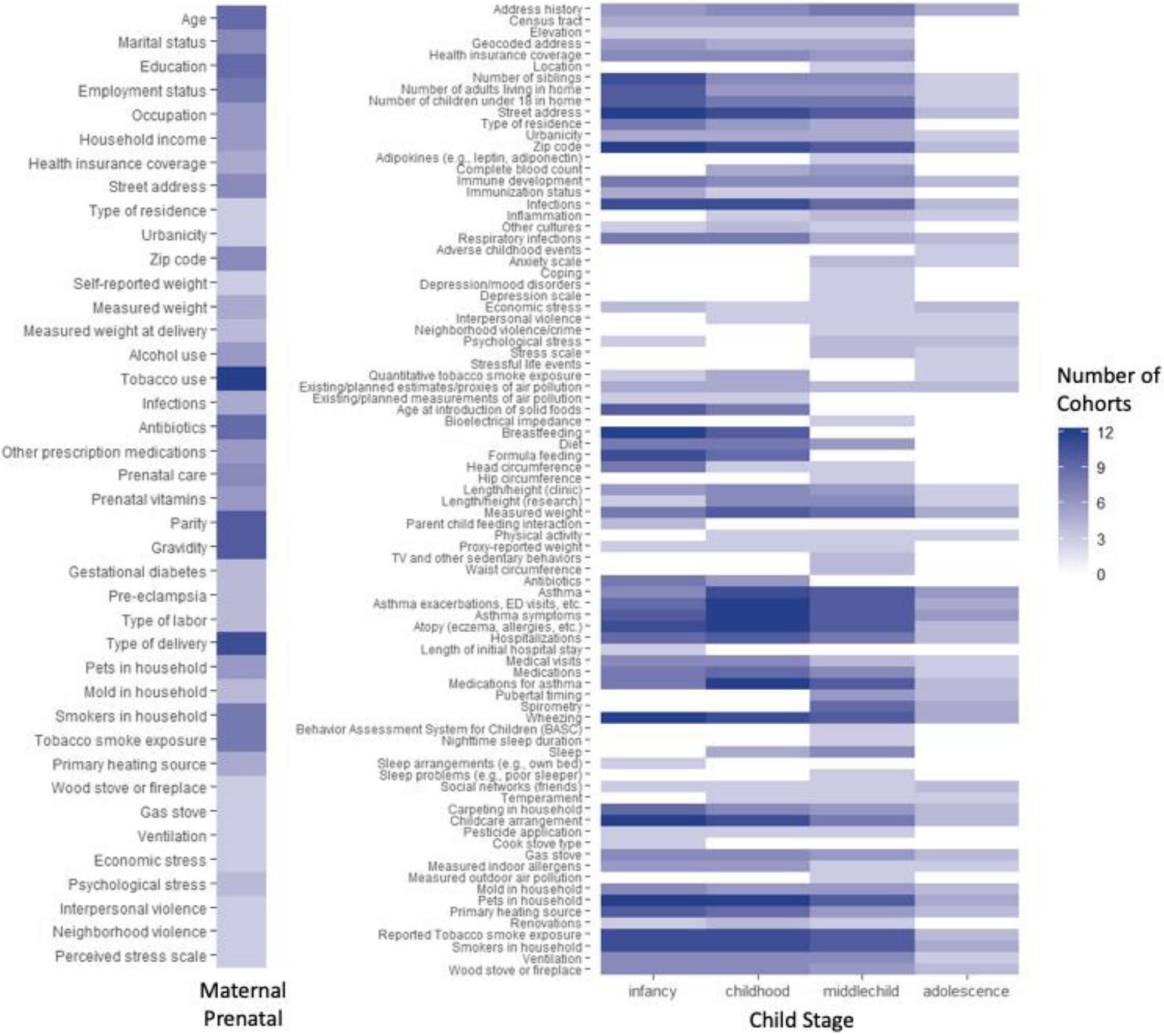
Data elements common to multiple cohorts. The number of cohorts that collected each data element is shown according to maternal prenatal exposure (**a**) and childhood life stage (**b**); infancy 0–11 mo., early childhood 1–4 years, middle childhood 5–11 years, adolescence 12–18 years)

The ECHO Data Analysis Center (Research Triangle Inc. and Johns Hopkins University) and CREW Biostatistics and Bioinformatics Core have surveyed the CREW member cohorts to identify common data elements to help direct the development of collaborative studies, listed by age group in Fig. 2. All cohorts have collected address information that will be translated into geocodes to be used to estimate a broad variety of exposures. During infancy and early childhood, multiple cohorts collected information on exposures to diet, allergens, tobacco smoke and pets. Immunologic testing was conducted in 11 cohorts in early life. Other areas of common data collection included early life viral respiratory infections (8 cohorts), allergen exposure (8 cohorts), obesity (8 cohorts), microbial exposures and/or colonization (7 cohorts) and psychosocial stress (5 cohorts). All cohorts collected information on allergic outcomes and asthma.

#### Geocoding

All 12 study cohorts completed distributive analyses to link addresses to geocodes. A total of 8978 birth record addresses were available for participants enrolled in CREW cohorts and, of these, 98.1% (*n* = 8810) were geocoded with sufficient precision for additional analyses. The CREW workgroup has also worked closely with the ECHO Data Analysis Center (Johns Hopkins University and RTI) to apply our approach to the entire ECHO consortium to facilitate similar analyses across all ECHO cohorts.

Future work in CREW will extend these methods to additional spatiotemporally variable physical (e.g. air pollution, greenspace, temperature) and social exposures. In addition, we will link the derived environmental exposures and community characteristics to longitudinal wheeze phenotypes, asthma, and other childhood respiratory health outcomes to examine the associations of environmental exposures and community characteristics with these outcomes.

#### Inventory of samples available for analysis

**Fig. 3:**
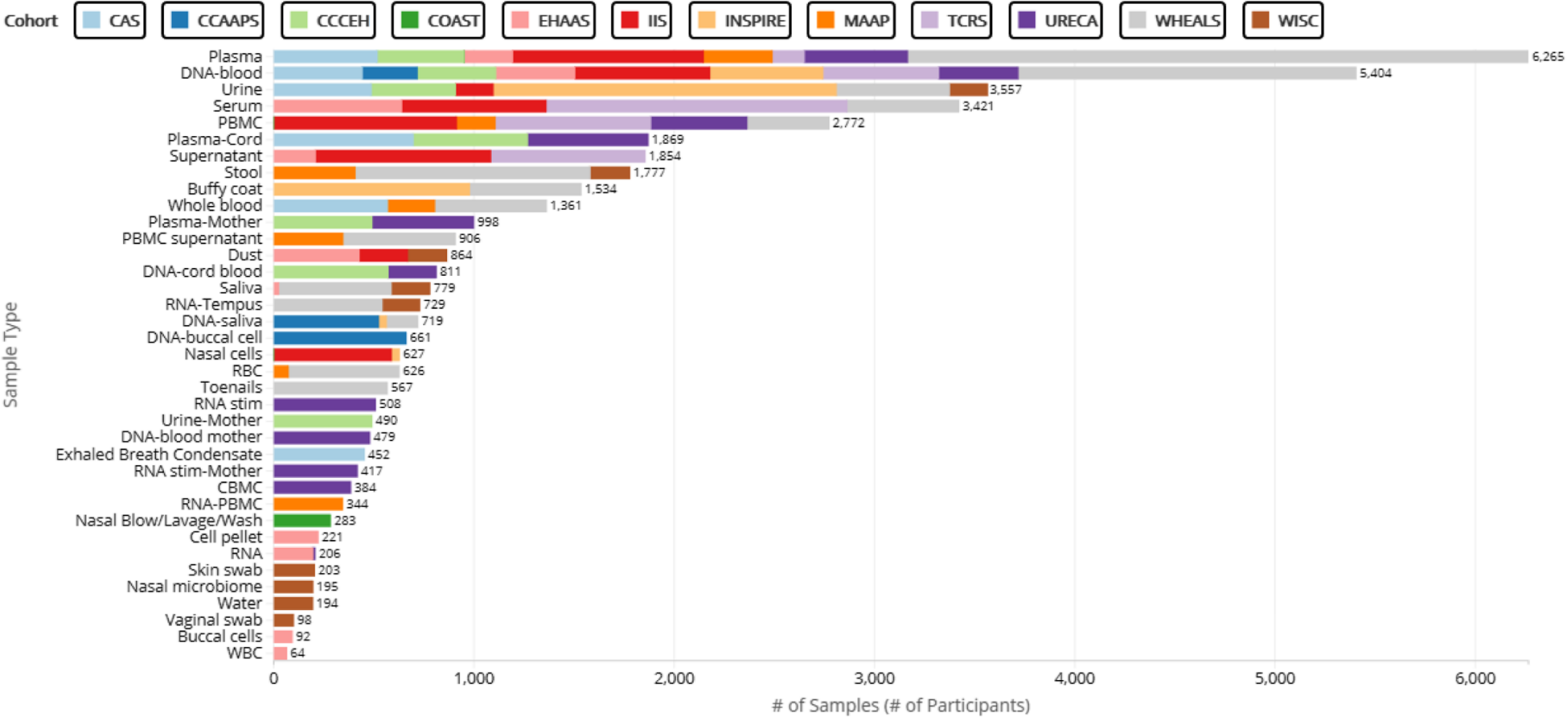
Samples collected by birth cohorts participating in the CREW consortium. The types of samples are indicated by the row titles, and the number of cohort-specific samples are color coded

An inventory of stored samples (including serum, cells, DNA) in CREW cohorts has been completed. An explorer tool has been developed by the CREW Coordinating Center to enable CREW investigators to interactively query available samples by sample type, cohort, and age of child at collection (Fig. 3). This tool will be used to identify samples that can be used for “omics” analyses, allergic outcomes (allergen-specific IgE), biomarkers and exposure assessments. Importantly, this will provide an excellent resource for identifying sample sets for collaborative ECHO analyses.

#### Analysis projects and publications

One of the main goals of CREW is to identify respiratory phenotypes based on longitudinal patterns of wheeze and other variables closely related to asthma such as allergic sensitization and lung function. Initial phenotyping of study participants based on longitudinal analysis of patterns of wheezing illnesses through the first decade of life is in progress. Analysis projects can be proposed by any CREW investigator, and are then reviewed and approved by the Publications and Steering Committees. Investigators from each cohort then have the final decision as to whether to participate in an analysis proposal. The ten currently approved analysis projects are listed in Table 5.

Because there is no universally accepted “gold standard” definition for the diagnosis of asthma in early childhood and definitions varied by study, we are exploring several different ways to classify asthma, including parental report of doctor-diagnosed asthma, and doctor-diagnosed asthma with either symptoms or controller therapy in the past 12 months, which can work across all our cohorts.

**Table 5:** Approved CREW analysis proposals

Title	Participating Cohorts												
	CAS	CCAAPS	CCCEH	COAST	EHAAS	IIS	INSPIRE	MAAP	TCRS	URECA	WHEALS	WISC	Total
Childhood research definitions of asthma outcomes	X	X	X	X	X	X			X	X	X		9
The natural history of pediatric asthma	X	X	X	X	X	X			X	X	X		9
Role of CDHR3 and 17q21 genetic variants as determinants of wheezing phenotypes in early life	X	X	X	X	X	X	X	X	X	X	X	X	12
Metabolomic profiling to identify pathways and endotypes of atopic and non-atopic childhood wheeze							X	X				X	3
Estimating wheezing severity in young children using a latent variable approach with the ISAAC wheezing module*					X		X						2
Vertical transmission of vaginal microbiota to the infant gut								X				X	3
Fine mapping of the 17q21 region and asthma	X	X	X	X	X		X		X	X	X		10
Regional and individual level socioeconomic characteristics of the CREW consortium	X	X	X	X	X	X	X	X	X	X	X	X	12
RSV and RV respiratory viral wheezing illness and 17q12–21 genotype				X			X		X	X		X	5

*Published [32]

### Phase II: standardized data collection

Participants who are currently enrolled or were previously enrolled in 10 of the CREW cohorts are being invited to participate in the standardized prospective CREW visits. The response by study participants has generally been positive, with < 5% refusals overall and 78% of the recruitment target being reached. Study visits began in August 2018, and the schedule of activities is shown in Table 3.

### Phase III: NewCREW

The NewCREW phase III protocol that will enroll approximately 400 additional pregnant women and their infants during the prenatal period at four CREW centers is being drafted. Enrollment is scheduled to begin in 2019.

## Discussion

The CREW consortium is integrating data from 12 US birth and infant cohort studies to identify prenatal and early life environmental factors that modify the risk for developing allergic diseases and asthma. These cohorts represent a number of US demographics, with good representation of white, African American and Hispanic children and their families. Each cohort has previously identified risk factors for the development of allergies and asthma and provided insights into asthma pathogenesis. Pooling data from individual cohorts to create the CREW cohort presents further opportunities, including the ability to test for risk factors for less common but clinically important outcomes such as severe childhood asthma, combined effects of multiple risk factors, geographical differences and mediating effects. Recruitment for some cohorts started in the 1980s while others are still recruiting today, and the several decade time span of study participation will enable us to test whether the risk factors for childhood asthma have changed over time in concert with marked increases in asthma prevalence. Notably, childhood asthma is a syndrome consisting of multiple asthma phenotypes that are difficult to discern using cross-sectional observations. By assessing a large and diverse cohort over time, the ultimate goal of CREW is to identify phenotypes of asthma and determine whether there are specific relationships between prenatal and early life risk factors that contribute to individual asthma phenotypes.

While this is the first US birth cohort consortium, several European consortia have been established to compare study designs and findings, standardize data collection, and perform meta-analyses or pooled analyses from multiple individual cohorts [28]. Examples include the Global Allergy and Asthma European Network (GA^2^LEN, established 2004) which brought together 25 research institutes in 16 countries to assess exposure variables, and allergic and respiratory outcome parameters [29]. Several other consortia have formed since 2009 to combine resources of multiple cohorts. These include Environmental Health Risks in European Birth Cohorts (ENRIECO), the Developing a Child Cohort Research Strategy for Europe (CHICOS) consortium, the Mechanisms of the Development of ALLergy (MeDALL), and the Study Team for Early Life Asthma Research (STELAR). CREW represents a unique opportunity to pool data from US cohorts. While productivity from individual cohorts has been high in the US, infrastructures to support collaborative research among allergy and asthma birth cohorts have been lacking. In recognition of this limitation, the NIAID and NHLBI sponsored the Birth Cohorts in Asthma and Allergic Diseases workshop in 2013 together with investigators from the MeDALL consortium [30]. This workshop developed strategies for harmonization of existing asthma birth cohort data, discussed study designs and standardized methods for new data collection, and identified areas of unmet needs. The conference attendees, which included many current CREW and ECHO investigators, concluded that “establishing criteria for asthma phenotypes that are mechanistically-based and reflect biological entities, including degrees of severity, is the highest priority”. The CREW consortium is designed to address this knowledge gap by pooling existing data, enriching these data through additional analyses on stored samples, and by enrolling new families into a protocol designed to optimize sampling procedures for systems analysis (genetics and epigenetics, microbial genomics, transcriptomics and metabolomics). Our goal is to analyze these data sets using predictive modeling techniques to identify endotypes of asthma related to specific environmental and personal risk factors. Pooling of existing data sets within CREW rather than performing meta-analyses should be helpful with respect to ease of analysis, greater statistical power, and availability of advanced statistical techniques that require a single data set.

CREW cohorts have additional opportunities for collaborative research within the U.S. and internationally. As a component of ECHO, standardized questionnaires and data elements have been developed to measure early life exposures that could affect multiple aspects of child health (perinatal outcomes, neurocognitive development, obesity and respiratory health). In addition, ECHO includes an integrated outcome entitled “positive health”, which incorporates measures of physical, mental and social wellbeing. Detailed assessment of these outcomes will enable CREW investigators to test for interactions among these health conditions and allergies and asthma in a large sample size (estimated 50,000 participants). Approximately two thirds of ECHO cohorts have prospectively collected information about respiratory outcomes, and all will complete standardized allergy and respiratory outcome assessments. In addition, about a quarter of the cohorts had focused on asthma prior to ECHO, and the ECHO Airways Outcome Working Group has a rich extant data set of predictors and outcome assessments for allergic diseases and asthma. Furthermore, adaptation and collaborative development of the STELAR Asthma eLab for use in CREW will promote research efforts between US and European cohorts.

Limitations of the CREW Consortium are mainly related to the different study designs and methods used to collect early life data. As a result, data will need to be harmonized to enable pooling for group analyses. Because the majority of the children are school-aged or older, most of the early life data in CREW already have been collected. Nonetheless, some opportunities exist for enriching data sets with the use of retrospective questionnaires. For example, retrospective analyses of pet ownership during early childhood correlates well with prospectively collected data [31]. In addition, use of time-adjusted geospatial technology will enable enrichment of early life exposure data related to pollutants and a wide range of social and economic variables.

In conclusion, CREW represents the first large scale collaborative study involving U.S. birth cohorts focused on allergic diseases and asthma in children. Pooling of extant data in CREW and using standardized outcomes will provide many opportunities to identify early life environmental exposures that are linked to specific endotypes of asthma. As part of the ECHO program, CREW will also have access to data from a much larger sample size and be able to ask questions about the relationship between asthma and other health outcomes in children.

## Data Availability

The datasets used during the current study are available from the corresponding author on reasonable request.

## Abbreviations

BBC: Biostatistics/Bioinformatics core
CAS: Childhood Asthma Study
CHICOS: Developing a Child Cohort Research Strategy for Europe
COAST: Childhood Origins of Asthma Study
CREW: Children’s Respiratory and Environmental Workgroup
CRF, eCRF: Case report form, electronic case report form
ECHO: Environmental influences on Child Health Outcomes
EHAAS: The Epidemiology of Home Allergens and Asthma Study
eNO: Exhaled nitric oxide
ENRIECO: Environmental Health Risks in European Birth Cohorts
FHIR: Fast Health Interoperability Resources
GA^2^LEN: Global Allergy and Asthma European Network
HIPAA: Health Insurance Portability and Accountability Act
INSPIRE: Infant Susceptibility to Pulmonary Infections and Asthma following RSV Exposure
ISAAC: International Study of Asthma and Allergies in Children
MAAP: Microbes, Allergy, Asthma and Pets
MDI: Metered dose inhaler
MeDALL: Mechanisms of the Development of ALLergy
NHLBI: National Heart Lung and Blood Institute
NIAID: National Institute for Allergy and Infectious Disease
PHI: Protected health information
STELAR: Study Team for Early Life Asthma Research
UW: University of Wisconsin
WHEALS: Wayne County Health, Environment, Allergy and Asthma Longitudinal Study
WISC: Wisconsin Infant Study Cohort
